# Longstanding Hyperthyroidism Is Associated with Normal or Enhanced Intrinsic Cardiomyocyte Function despite Decline in Global Cardiac Function

**DOI:** 10.1371/journal.pone.0046655

**Published:** 2012-10-04

**Authors:** Nathan Y. Weltman, Dajun Wang, Rebecca A. Redetzke, A. Martin Gerdes

**Affiliations:** 1 Department of Basic Biomedical Sciences, Sanford School of Medicine at the University of South Dakota, Sioux Falls, South Dakota, United States of America; 2 Cardiovascular Research Center, Sanford Research/University of South Dakota, Sioux Falls, South Dakota, United States of America; 3 Department of Biomedical Sciences, New York College of Osteopathic Medicine at New York Institute of Technology, Old Westbury, New York, United States of America; Ohio State University, United States of America

## Abstract

Thyroid hormones (THs) play a pivotal role in cardiac homeostasis. TH imbalances alter cardiac performance and ultimately cause cardiac dysfunction. Although short-term hyperthyroidism typically leads to heightened left ventricular (LV) contractility and improved hemodynamic parameters, chronic hyperthyroidism is associated with deleterious cardiac consequences including increased risk of arrhythmia, impaired cardiac reserve and exercise capacity, myocardial remodeling, and occasionally heart failure. To evaluate the long-term consequences of chronic hyperthyroidism on LV remodeling and function, we examined LV isolated myocyte function, chamber function, and whole tissue remodeling in a hamster model. Three-month-old F1b hamsters were randomized to control or 10 months TH treatment (0.1% grade I desiccated TH). LV chamber remodeling and function was assessed by echocardiography at 1, 2, 4, 6, 8, and 10 months of treatment. After 10 months, terminal cardiac function was assessed by echocardiography and LV hemodynamics. Hyperthyroid hamsters exhibited significant cardiac hypertrophy and deleterious cardiac remodeling characterized by myocyte lengthening, chamber dilatation, decreased relative wall thickness, increased wall stress, and increased LV interstitial fibrotic deposition. Importantly, hyperthyroid hamsters demonstrated significant LV systolic and diastolic dysfunction. Despite the aforementioned remodeling and global cardiac decline, individual isolated cardiac myocytes from chronically hyperthyroid hamsters had enhanced function when compared with myocytes from untreated age-matched controls. Thus, it appears that long-term hyperthyroidism may impair global LV function, at least in part by increasing interstitial ventricular fibrosis, in spite of normal or enhanced intrinsic cardiomyocyte function.

## Introduction

Thyroid hormones (THs) play a pivotal role in regulating cardiac homeostasis as well as the peripheral vascular system in physiologic and pathologic conditions [1], [2]. THs influence heart rate (HR), myocardial contractility, total peripheral resistance (TPR), and ultimately cardiac output. At the cellular level, THs enhance myocardial contractility by regulating the expression of Ca^2+^ handling, myosin heavy chain isoforms (β→α), and potentiating the β-adrenergic system [1], [3], [4]. THs also exert their influence by regulating non-myocyte cells such as fibroblasts, vascular smooth muscle cells, pericytes, and adipocytes.

Excess TH is associated with elevated HR, decreased TPR, widened pulse pressure, blood volume expansion, and increased cardiac output [1]. In the short term, hyperthyroidism is associated with heightened left ventricular (LV) contractile function and improved hemodynamic parameters. However, excess TH levels increase tissue metabolic rate, ATP consumption, and heat production, which ultimately leads to increased peripheral oxygen consumption, inefficient myocardial energy utilization, and increased cardiac work [5]–[7]. The consequences of sustained hyperthyroidism include increased risk of arrhythmias, impaired cardiac reserve and exercise capacity, and myocardial remodeling [8]–[12]. Longstanding hyperthyroidism leads to cardiac impairment characterized by low cardiac output, chamber dilation, and “heart failure like” symptoms [13]–[18]. Interestingly, the dilation and diminished cardiac function caused by thyrotoxicosis often is ameliorated or reversed when euthyroidism is re-established.

A better understanding of the progression and cellular mechanisms responsible for cardiac dysfunction during periods of sustained hyperthyroidism is clinically important. There is limited information within the current literature examining the relationship between myocyte function and global cardiac function during the transition from cardiac compensation to decompensation in the setting of sustained hyperthyroidism. Furthermore, there is limited and conflicting information regarding the functional consequences of increased LV fibrotic deposition in the setting of sustained hyperthyroidism. While previous investigations have examined the influence of hyperthyroidism on cardiac function either *in vivo* or *in vitro*, the relationship between *in vivo* cardiac function, *in vitro* isolated myocyte function, and LV fibrosis in this setting is poorly understood. Our lab previously characterized the influence of hyperthyroidism on cardiac remodeling and function during short (10 days) and moderate length (2 months) treatment periods in F1B hamsters [19].

To provide better understanding of the long-term consequences of chronic hyperthyroidism on LV remodeling and function, we examined global cardiac function, LV isolated myocyte function, and whole tissue remodeling using the previously characterized F1B hamster model. This study suggests that the impairment in overall cardiac function observed with long standing hyperthyroidism is not related to decline in the functional capacity of individual myocytes.

## Materials and Methods

### Animal Model and Experimental Design

The use of animals in this study conformed to the Public Health Service Guide for Care and Use of Laboratory Animals. All experiments and protocols were approved by the Sanford Research/USD Institutional Animal Care and Use Committee. Three month old BIO F1B hamsters (BIO Breeders INC., Watertown, MA) were randomized to either TH (hyperthyroid) or untreated (control) groups. Hamsters in the TH treatment group were fed pellets containing 0.1% grade I desiccated TH (Sigma # T1251) as previously described [19]. All animals were kept on a 14 hour light/10 hour dark cycle and food and water were provided *ad libitum*. LV chamber remodeling and function was assessed by serial echocardiography at 1, 2, 4, 6, 8, and 10 months of treatment. Terminal echocardiography and LV hemodynamic assessment of function were collected after 10 months of TH treatment. Following terminal experiments, animals were randomized to either cell isolation to evaluate individual myocyte performance or whole heart tissue collection. Prior to sacrifice, animals were injected with heparin (1000 U/kg, i.p.). Whole hearts were arrested in diastole by injecting an ice cold solution containing 0.2% 2, 3-butanedione monoxine and 0.1% heparin dissolved in PBS into the LV. Hearts were then quickly removed, rinsed in ice cold PBS, blotted and weighted. LVs were dissected, weighed, and sectioned transversely and fixed in ice cold 10% formalin or embedded in OCT compound (Sakura Finetek Inc., Torrance, CA) and frozen in liquid nitrogen.

### Measurement of Serum Thyroid Hormones

Terminal LV blood samples were collected and separated into serum aliquots by centrifugation and stored at −80°C. T_3_ and T_4_ levels were measured using commercial ELISA kits as previously described [20].

### Echocardiography

Echocardiography was performed in each animal at 1 month, 2 months, and then every two months until terminal experiments using a Vevo 660 high-resolution imaging system with a 25-MHz RMV-710 transducer (Visualsonics; Toronto, Canada) as previously described [21]. Briefly, hamsters were anesthetized using isoflurane (1.5%) and two-dimensional echocardiograms were obtained from short-axis views of the left ventricle (LV) at the level of the papillary muscle tips. Two dimensional M-mode echocardiograms were used to measure LV dimensions in systole and diastole.

### LV Hemodynamic Measurements

Prior to sacrifice, LV hemodynamics and aortic pressures were obtained by catheterization of the right carotid artery using a Millar Micro-tip catheter (Millar Instruments; Houston, TX) as described previously [22]. After stabilization, LV and aortic measurements were recorded electronically using Chart 5 software (ADInstruments Inc., Colorado Springs, CO). LV meridional wall stress was estimated using previously reported methods [23].

### LV Myocyte Isolation

LV myocyte isolation was adapted from previously described methods [24], [25]. After whole heart excision, the aorta was cannulated and perfused with 37°C Krebs Henseleit Bicarbonate (KHB) buffer containing (in mM): 113 NaCl, 4.7 KCl, 0.6 KH_2_PO_4,_ 0.6 Na_2_HPO_4_, 1.2 MgSO_4_-7H_2_0, 10 Na-Hepes, 12 NaHCO_3_, 30 Taurine, 10 BDM, 5.5 Glucose. Hearts were subsequently perfused with 225 U/ml collagenase type II (Worthington Biochemical, Lakewood, NJ) in KHB solution. The LV was dissected, minced, filtered through a nylon mesh, and centrifuged in stopping buffer (10% bovine calf serum, 12.4 mM CaCl_2_ in KHB). Re-suspended cells were transferred to a dish containing ATP (final conc. 2 mM). CaCl_2_ was then slowly added back until a final concentration of 1.25 mM was reached.

### Isolated Cell Mechanics

After 10 months TH treatment, isolated rod-shaped myocytes with discernible striations and clear edges were evaluated using an IonOptix myocyte contractility system (IonOptix Corp., Milton, MA). Myocytes were placed in a chamber mounted on the stage of an inverted microscope (Nikon Eclipse TS300; Melville, NY) and superfused with sterile filtered, 37°C Tyrode’s buffer containing (in mM): 137 NaCl, 5.4 KCL, 1.2 CaCl_2_, 1.5 MgCl_2_, 10 HEPES, 10 glucose, pH 7.4. Myocytes were field stimulated with suprathreshold voltage (+20%) at a frequency of 0.5 Hz, 3 msec duration, using platinum wires placed on opposite sides of the chamber connected to an acute field stimulator. Polarity of the stimulatory electrodes was frequently reversed to avoid buildup of electrolysis byproducts. Myocytes were displayed using an Ionoptix MyoCam camera and edge detection software (IonOptix Corp).

### Histology

Transverse 5 µm LV tissue sections were stained with Masson’s trichrome or haematoxylin and eosin. Myocardial fibrosis was visualized at 10× and quantified using Image J software (NIH, Bethesda, MD; http://rsbweb.nih.gov/ij/). Values are represented as the proportion of collagen normalized to the total solid tissue area to minimize any variation due to tissue separation.

### Data Analysis

All data are expressed as means ± (S.D.). Diagnostics were conducted to verify assumption of normality and variance before applying the models. Statistical analysis was performed using a two-tailed Student’s T-test or Mann-Whitney rank sum test. Values of p<0.05 were considered statistically significant. Statistical analysis was performed using Sigmastat V 3.5 (Aspire Software International; Ashburn, VA).

## Results

### Physical Data and TH Levels

Physical data and serum TH levels are presented in Table 1. There was no difference in baseline body weight (BW) between control and TH treated hamsters [data not shown]. By one month, TH treatment resulted in a significant and sustained BW increase [Table 1; 10 month data shown]. Treatment led to significant cardiac hypertrophy as indicated by increased heart weight (HW) and ∼20% higher HW/BW ratio. Our findings of increased BW, HW, and HW/BW ratio are consistent with previous reports [19], [26]. As expected, treated hamsters had significant elevations in serum T3 and T4 levels.

**Table 1:** Physical characteristics and serum thyroid hormones.

	Control	Hyperthyroid	p-Value
**BW (g)**	171 (11)	185 (13)	0.01
**HW (mg)**	597 (48)	804 (94)	<0.001
**HW/BW (mg/g)**	3.51 (0.3)	4.38 (0.6)	<0.001
**T3 (ng/ml)**	0.6 (0.2)	3.8 (0.9)	<0.001
**T4 (µg/dl)**	4.8 (1.0)	13.9 (2.7)	<0.001

Values are means (SD). BW, body weight; HW, heart weight; HW/BW, heart weight to body weight ratio. N = 12−15/group.

### Prolonged Hyperthyroidism Causes Adverse Chamber Remodeling and Decline in LV Function as Assessed by Echocardiography

**Figure 1:**
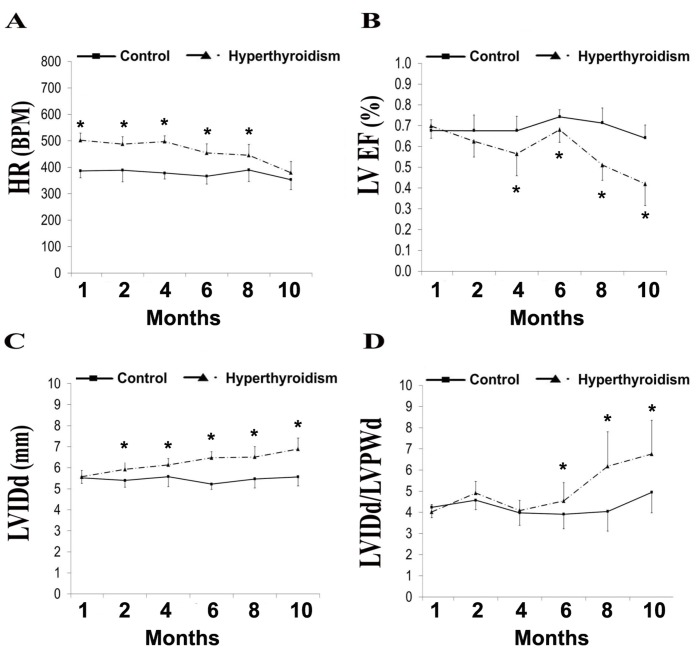
Temporal echocardiographic changes. Values are means (SD). A–D. HR, heart rate (A); LV EF, left ventricular ejection fraction (B); LVIDd, left ventricular internal dimension in diastole (C), LVIDd/LVPWd ratio (D). N = 13−15/group. *, p<0.05 vs. control.

By one month, TH treatment resulted in a significant elevation in resting HR. Tachycardia was sustained during the initial 8 months of treatment. Thereafter, HR declined to control levels [Fig. 1A]. By 4 months, there was significant depression of ejection fraction (EF) in the treated group. Between 6 and 10 months of treatment, there was a severe and progressive reduction (∼10% at 6 months; ∼30% at 8 months; ∼36% at 10 months vs. control) in measured LV EF [Fig. 1B].

There was no difference in LV internal dimension during diastole (LVIDd) after 1 month TH treatment. However, treated hamsters had significant increases in LVIDd by two months [Fig. 1C]. Initially, this increase was compensated for by a relative increase in LV posterior wall thickness (LVPWd). Despite the relative preservation of LVPWd, progressive chamber enlargement (LVIDd) led to significant elevations in the anatomical parameters of diastolic wall stress as indicated by an increased LVIDd/LVPWd ratio by 6 months [Fig. 1D]. Interestingly, declines in LVPWd were not observed until 8 months of TH treatment (∼21% reduction, non-significant). This “relative” wall thinning led to a significant increase in LVIDd/LVPWd after 8 months of hyperthyroidism. Terminal invasive studies confirmed that TH treated hamsters had significantly elevated meridional wall stress both at end-diastolic and end-systolic [Table 2].

**Table 2:** LV hemodynamics.

	Control	Hyperthyroid	p-Value
**SBP (mmHg)**	156 (15)	134 (12)	<0.002
**DBP (mmHg)**	84 (12)	75 (16)	0.20
**LV ESP (mmHg)**	160 (16)	123 (11)	<0.001
**LV EDP (mmHg)**	8 (5)	12 (6)	0.138
**dP/dT Max (mmHg/sec)**	9921 (1980)	7291 (708)	<0.001
**dP/dT Min (mmHg/sec)**	−8998 (1844)	−4844 (683)	<0.001
**Tau (msec)**	11 (4)	15 (5)	0.004
**Wall Stress (ED), kdyne/cm^2^**	12.8 (7)	26.2 (12)	0.005
**Wall Stress (ES),** **kdyne/cm^2^**	137.7 (32)	194.5 (33)	<0.001

Values are means (SD). SBP, systolic blood pressure; DBP, diastolic blood pressure; LV ESP, left ventricular end systolic pressure; LV EDP, left ventricular end diastolic pressure; dP/dT Max, maximal rate of pressure development; dP/dT Min, maximal rate of pressure decline; Tau, time constant of left ventricular isovolumic relaxation; Wall Stress ED, wall stress at end diastole; Wall Stress ES, wall stress at end systole; Meridional Wall stress calculated using previously described methods [23]. N = 12−13/group for all measurements except SBP, DBP (N = 9 & 11 in control and treated, respectively) and wall stress (N = 11 & 10 in control and treated respectively).

### Sustained Hyperthyroidism Causes Decline in LV Hemodynamic Parameters

Compared with age-matched control hamsters, sustained hyperthyroidism was associated with impaired LV function [Table 2]. Treated hamsters had significant declines in systolic blood pressure, LV end systolic pressure, dP/dT max (maximum rate of pressure rise), dP/dT Min (maximum rate of pressure decline), and increased Tau (time constant of isovolumic relaxation). Diastolic blood pressure and LV end diastolic pressure were not significantly affected by TH treatment.

### Isolated Myocytes from Hyperthyroid Hamsters have Enhanced Mechanical Function

**Figure 2:**
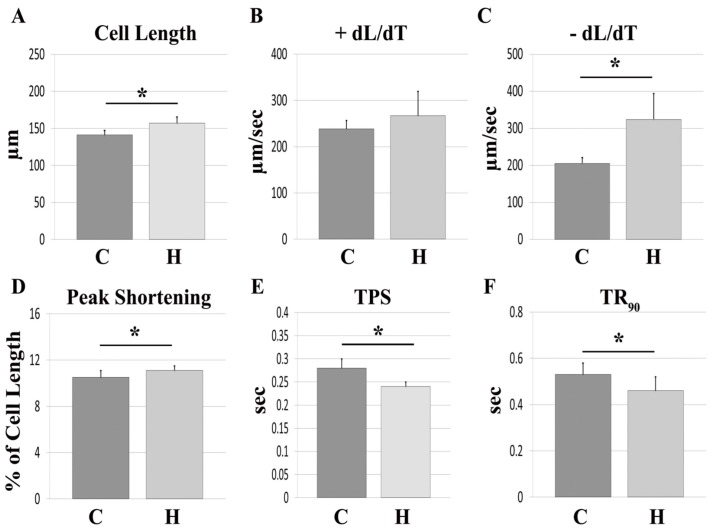
Isolated cardiomyocyte mechanics following 10 months of thyroid hormone treatment. Values are means (SD). A–F. Cell Length (A); +dL/dT, maximal velocity of shortening (B); –dL/dT, maximal velocity of re-lengthening (C); Peak Shortening (D); TPS, time to peak shortening (E), TR_90_, Time to 90% re-lengthening (F). C, control; H, hyperthyroid. N = 5−7/group. *, p<0.05 vs. control.

Despite global cardiac impairment observed by echocardiography and LV hemodynamics, treatment improved functional mechanics of individual isolated myocytes [Fig. 2]. Treatment was associated with significant improvement in – dL/dT (maximum velocity of re-lengthening), peak shortening, time to peak shortening, and time to 90% re-lengthening. Peak Velocity of Shortening (+dL/dT) was not significantly affected. Treated hamsters had increased resting myocyte length.

### Sustained Hyperthyroidism is Associated with Increased LV Fibrosis

**Figure 3:**
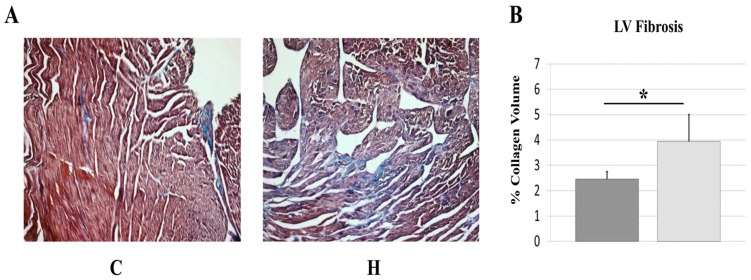
LV fibrosis. Values are means (SD). Representative images of LV fibrosis stained by Masson’s Trichrome (A). % collagen volume as a proportion of total tissue area (B). C, Control; H, Hyperthyroid. N = 5/group. *, p<0.05 vs. control.

Representative images of LV fibrosis are depicted in Fig. 3A. Compared with untreated hamsters, treatment was associated with ∼60% increase in LV fibrosis [Fig. 3B]. The observed increase in LV collagen was primarily located within the perivascular space (perivascular fibrosis) and cardiac interstitium (interstitial fibrosis). Myocyte necrosis with replacement fibrosis was not observed.

## Discussion

The major finding of this study is the development of a mismatch between global cardiac performance and individual myocyte function in the setting of chronic hyperthyroidism. Chronic hyperthyroidism was associated with deleterious cardiac remodeling characterized by myocyte lengthening, chamber dilatation, decreased relative posterior wall thickness, increased wall stress, and increased LV fibrotic deposition. Importantly, sustained hyperthyroidism led to LV systolic and diastolic dysfunction as evaluated by echocardiography and LV hemodynamics. The novel feature of this study is the finding that despite global cardiac impairment, individual isolated cardiac myocytes from chronically hyperthyroid hamsters had enhanced mechanical function when compared with myocytes from untreated age matched controls. This paradox suggests that the cardiac dysfunction observed during prolonged hyperthyroid conditions is not the consequence of declined functional capacity of individual myocytes, but rather impairment in the ability of individual myocytes to function properly in the whole tissue setting.

It has long been recognized that THs plays a pivotal role in cardiovascular homeostasis [1]. Excess THs cause a hyperdynamic state characterized by decreased TPR and blood volume expansion, which in turn can lead to augmented contractility and increased cardiac output [1], [5]. Protracted hyperthyroidism is associated with deleterious cardiac changes as the consequence of increased hemodynamic burden. This was elegantly shown by Klein and Hong using heterotopic cardiac transplanted hearts [27]. They were able to demonstrate that cardiac hypertrophy which often accompanies TH excess is an indirect consequence of the superimposed hemodynamic burden and resultant increased cardiac work rather than a direct effect of TH on the cardiac tissue.

In an attempt to maintain cardiac function and compensate for increased cardiac stress during prolonged TH excess, the heart undergoes a remodeling process characterized by myocyte enlargement and extracellular matrix (ECM) deposition. The arrangement of myocytes within the ventricular wall is such that cell length is primarily responsible for chamber diameter and myocyte cross sectional area (CSA) is responsible for changes in wall thickness [28]. During the initial phases of hyperthyroidism, compensation occurs by proportional growth in both myocyte length and CSA [29]. However, the sustained hemodynamic load eventually overwhelms the compensatory ability of the heart and causes progression to a dilated ventricle characterized by chamber dilation (↑ myocyte length) without further increase in relative LVPW thickness (CSA). This maladaptive phenotype is characteristic of dilated heart failure (HF) and is associated with increased cardiac work, wall stress, and ECM deposition [28], [30], [31].

Our lab previously reported increased LV internal dimensions and chamber dysfunction after 2 months of hyperthyroidism in F1B hamsters [19]. In agreement with these findings, we observed progressive chamber dilation by 2 months of TH treatment [Fig. 1C]. In the present study, the LV continued to dilate until the time of terminal experiments in the hyperthyroid group. We have also shown that myocyte lengthening alone, due to series sarcomere addition, can account for chamber dilation in HF [28], [32], [33]. In the current study, myocyte lengthening observed in the hyperthyroid group is consistent with the increased LV internal dimension detected by echocardiography.

Despite the early increase in chamber internal dimension, a relative increase in LVPW thickness helped normalize the anatomical parameters of wall stress during the first 4 months of TH excess. By 6 months, hyperthyroid animals had a significantly elevated LVIDd/LVPWd ratio which steadily increased until the terminal 10 month time point. This progressive increase in the anatomical parameters of wall stress mirrored the decline observed in LV EF. Terminal invasive measurements confirmed that treated animals had significant elevations in both end-diastolic (100% increase) and end-systolic (41% increase) meridional wall stress. Hyperthyroidism initially resulted in tachycardia, however it is important to note that HR declined to control levels by the end of the study when LV dysfunction was most pronounced. This reduction of TH-induced tachycardia observed after 8 months likely represents the onset of adrenergic decompensation. Tachycardia is a widely used diagnostic marker in the identification of hyperthyroidism. Our findings suggest that HR may not always be a reliable predictor of hyperthyroidism, especially in the setting of advanced cardiac disease caused by sustained TH excess.

To our knowledge, this is the first report of a paradoxical mismatch between global cardiac function and individual myocyte function in the setting of prolonged hyperthyroidism. Several previous reports lend credence to the idea that global cardiac function is not a consistent indicator of individual myocyte contractile function [34]–[39]. Although the exact etiology of this discrepancy is unknown, several myocyte and non-myocyte factors likely contribute. Alterations in excitation-contraction coupling, Ca^2+^ handling properties, neurohumoral activation, oxidative stress, vascularity and blood flow, cell metabolism, cell death (apoptosis or necrosis), fibrotic deposition, and myocyte remodeling have all been implicated. While we cannot exclude the aforementioned parameters as contributing to the discrepancy, myocyte necrosis or apoptosis appear unlikely. Areas of cell loss and replacement fibrosis were not observed, reducing the likelihood of myocyte necrosis. Except with extreme changes, such as in the peri-infarct area after acute myocardial infarction, apoptosis appears to predominantly occur in non-myocytes during HF and cardiac dysfunction [40]. When myocyte loss occurs by apoptosis, fibrous deposition/replacement is not present and would be difficult to document over such a long treatment period [41]. Based on tissue morphology and the fact that THs tend to inhibit apoptosis [42], there is little reason to suspect that apoptosis accounts for significant loss of contractile cells or fibrotic deposition in the current setting. Although we cannot exclude the possibility of diminished coronary blood flow, it is unlikely in the current experimental setting. THs are potent stimulators of coronary angiogenesis and blood flow in the setting of hyperthyroidism. THs have been shown to increase resting blood flow both with and without accompanying cardiac hypertrophy [43]–[45]. Furthermore, cardiac hypertrophy which is secondary to hyperthyroidism is typically associated with augmented blood flow and parallel or increased vascular growth due to increased cross-sectional area of the vascular bed [44], [46].

On the other hand, increased LV fibrosis and collagen crosslinking are associated with diastolic stiffness which contributes to LV pump dysfunction and progression to HF [31], [47]–[49]. While previous investigations have examined the influence of THs on collagen gene expression and/or fibrotic deposition, they typically have been performed without simultaneous assessment of cardiac function or are deduced from autopsy findings [13], [50]–[52]. For this reason, the influence of LV fibrosis on cardiac function during sustained hyperthyroidism is not well understood. THs have been implicated in the regulation of collagen dynamics, however, their influence on myocardial fibrosis has yielded conflicting results. Short-term studies have shown that THs exert anti-fibrotic actions, including in the setting of TH induced hypertrophy [6], [53]–[57]. *In vitro* investigations by Yao & Eghbali and Chen et al., suggest that THs can directly regulate and suppress collagen gene expression [53], [56]. In contrast, Roy et al. found that the anti-fibrotic actions of THs were the result of regulating matrix metalloproteinases (MMPs) and tissue inhibitors of metalloproteinases (TIMPs) despite increased collagen and pro-collagen gene expression [54]. On the other hand, myocardial fibrosis caused by chronic hyperthyroidism has been reported by numerous authors [7], [13], [58]–[60]. Chronic hyperthyroidism is associated with increased cellular metabolism and increased oxidative damage [61]–[64]. Oxidative damage is a known stimuli for collagen deposition [65]–[68] and increased oxidative damage which occurs during sustained bouts of hyperthyroidism likely contributes to increased interstitial collagen deposition. Further investigation is needed to fully elucidate the mechanisms of increased interstitial collagen deposition in this setting.

Although it is well established that acute hyperthyroidism is associated with augmented cardiac function and increased cardiac output [1], [5], [69], the importance of LV fibrosis on cardiac function during hyperthyroidism is not well characterized. Using trichrome staining, we were able to confirm in our study that prolonged hyperthyroidism was associated with increased LV fibrosis [∼60%↑] when compared with age matched control hamsters. The majority of fibrotic deposits were found within the perivascular space (perivascular fibrosis) and cardiac interstitium (interstitial fibrosis) without evidence of myocyte necrosis and replacement fibrosis. Moreover, we observed severe relaxation impairment (-dP/dT and Tau) and ultimately systolic dysfunction (LV EF, LV ESP, dP/dT) in hyperthyroid hearts. Our findings further support the notion that LV fibrosis inversely affects LV function in the setting of hyperthyroidism.

While increased collagen deposition certainly can impair global cardiac function, it may not similarly affect intrinsic cardiomyocyte mechanics. Accordingly, we also examined the influence of chronic hyperthyroidism on the mechanical function of individual myocytes. We hypothesized that mechanical impairment at the level of individual ventricular myocytes would strongly correlate with the decline observed in global cardiac function. Contrary to our hypothesis, we found that isolated ventricular myocytes from hyperthyroid hamsters had enhanced mechanical function when compared to age matched control hamsters, despite the aforementioned adverse chamber remodeling and diminished global cardiac function.

Given the close proximity of fibrillar collagen to myocytes and the finding that fibrillar collagen is a relatively stiff material with a tensile strength greater than steel [41], it is likely that even a small increase in collagen can impair cardiomyocyte function. Indeed, this notion is supported by reports that small changes in collagen concentration can have a profound impact on passive mechanical properties of cardiac tissue [31], [70]. In agreement, our findings suggest that relatively modest but significant increases in myocardial collagen deposition can impede myocyte contractile ability even when individual myocyte function is enhanced. Consistent with our previous report [19], we did not observe noticeable areas of fibronecrosis. This suggests that the depressed global cardiac function observed during chronic hyperthyroidism is at least in part the product of inhibited myocyte function caused by increased perivascular and interstitial fibrotic deposition and does not appear to be a product of extensive myocyte loss.

### Limitations

Our study has several limitations. While a standard experimental protocol was closely followed to replicate the same experimental conditions for each animal, it is possible that myocytes selected for functional assessment do not represent the total myocyte population within the intact heart. It is also important to note that isolated myocytes in the current study were not tested under loading conditions. Loading conditions can influence muscle function and altered loading theoretically could impact isolated myocyte shortening and tension development. Unfortunately, the technical difficulty of myocyte loading experiments limits its utility and widespread implementation [37], [71]. Unloaded isolated myocyte assessment still is an important tool for evaluating shortening velocity/cross bridge turnover rate and comparisons between treatment groups can be made under similar experimental conditions without influence of potential confounders. Finally, we acknowledge that comprehensive isolated myocyte morphometric analysis would have complemented the echocardiographic and isolated myocyte functional data presented in the current study. Although we are accustomed to high proportions of rod cell with our commonly used non-calcium tolerant myocyte preparation with rapid gluteraldehyde fixation (>90% rod cells are routine), the Ca^2+^ tolerant isolated myocyte preps used here did not produce high enough rod cell yields for accurate Coulter Channelyzer analysis.

### Conclusions

In summary, chronic hyperthyroidism was associated with deleterious cardiac remodeling, LV fibrosis, and cardiac functional decline. Despite global cardiac impairment, individual isolated cardiac myocytes from chronically hyperthyroid hamsters had normal or enhanced function when compared with myocytes from untreated age matched controls. While we cannot definitely establish a cause and effect relationship, our data strongly suggests that increased LV interstitial fibrosis can undermine the ability of otherwise normal myocytes to function properly. One can only speculate regarding translating ANY animal observations to humans. While chronic hyperthyroidism in humans is generally identified and treated before reaching this point, our results may provide an explanation for LV dysfunction observed in patients with chronic hyperthyroidism.
